# Supercapacitance/Resistance Behaviors of Helminth Eggs as Reliable Recognition and Direct Differentiation Probe

**DOI:** 10.3389/fbioe.2021.782380

**Published:** 2021-12-06

**Authors:** Ruhollah Shaali, Mohammad Mahdi Doroodmand, Mohammad Moazeni

**Affiliations:** ^1^ Department of Chemistry, Shiraz University, Shiraz, Iran; ^2^ Department of Pathobiology, School of Veterinary Medicine, Shiraz University, Shiraz, Iran

**Keywords:** helminth egg, supercapacitor, resistance, bio-recognition, bioelectronic, detection probe

## Abstract

Parasitic helminths are usually known as undesired pathogens, causing various diseases in both human and animal species. In this study, we explore supercapacitance/resistance behaviors as a novel probe for rapid identification and direct differentiation of *Fasciola hepatica*, *Parascaris equorum* (with and without larvae), *Dicrocoelium dendriticum*, *Taenia multiceps*, and *Moniezia expansa* eggs. This claim is attributed to some characteristics, such as grave supercapacitance/area, high-energy storage/area, large power/egg, huge permittivity, and great electrical break-down potential, respectively (Fasciola hepatica: 2,158, 0.485, 2.7 × 10^–3^, 267, 52.6, *Parascaris equorum* without larvae: 2,825, 0.574, 3.0 × 10^–3^, 351, 68.4, *Parascaris equorum* with larvae: 4,519, 0.716, 2.4 × 10^–3^, 1.96, 97.6, *Dicrocoelium dendriticum*: 1,581, 0.219, 2.8 × 10^–3^, 1.96, 48.8, *Moniezia expansa*: 714, 0.149, 2.2 × 10^–3^, 0.88, 35.2, *Taenia multiceps*: 3,738, 0.619, 4.7 × 10^–3^, 4.63, 84.4), and durable capacitance up to at least 15,000 sequential cycles at different scan rates (between 2.0 × 10^−4^ and 120.0 V s^−1^) as well as highly differentiated resistance between 400 and 600 Ω. These traits are measured by the “*Blind Patch-Clamp*” method, at the giga ohm sealed condition (6.18 ± 0.12 GΩ cm^−1^, *n* = 5). Significant detection ranges are detected for each capacitance and resistance with gradient limits as large as at least 880 to 1,000 mF and 400 to 600 Ω depending on the type of helminth egg. The effect of water in the structure of helminth eggs has also been investigated with acceptable reproducibility (RSD 7%–10%, *n* = 5). These intrinsic characteristics would provide novel facilitators for direct helminth egg identification in comparison with several methods, such as ELISA, PCR, and microscopic methods.

## Introduction

In the world of biosensors and bioelectronics, many biomaterials are considered as an essential target in the measurement or the heart of a measuring device (Willner and Katz, 2006). These include enzymes (Schneider and Clark, 2013), proteins (Leca-Bouvier and Blum, 2005), sugars (Zhang et al., 2013), lipoproteins (Lu et al., 2017), DNA (Zhang et al., 2013), and so on. In recent years, the use of these materials in identifying, measuring, and sensing other materials or even their use in the manufacture of the electronic components have been considered as one of the fields of interest to scientists (Ali et al., 2017; Sadighbayan et al., 2019). Due to the safety, availability, and their capability for amplification (Soleymani and Li, 2017), modification (Mittal et al., 2017), and miniaturization (Mowbray and Amiri, 2019), they are the aim of the researches.

On the other hand, the construction, research, and, development of bio-wearable devices in the field of energy storage in recent years have been considered as another exciting field of research (Choi et al., 2016). For instance, the use of high-capacitance materials to store energy is also considered (Zhang et al., 2017). These phenomena mainly attribute to the significant advances in material science during introducing synthetic compounds with reliable performances (Kim et al., 2015; Li et al., 2016). However, supercapacitors, as advanced materials in the field of energy storage, have been focused in recent years (Muzaffar et al., 2019). Up to now, several types of these materials have been introduced and categorized based on their different charge-storage mechanisms (Kim et al., 2015): “*Electrical Double Layer Capacitor*” (*EDLC*), “*Pseudo-Capacitors*,” “*Hybrid*,” and “*solid-state supercapacitor*” (Lu, 2013; Lu et al., 2014). These are convenient energy storage-conversion devices that often show rapid charging–discharging rates, with high-power density and long cycle life (Yu et al., 2013).

Up to now, scientists have focused on some different natural mineral and artificial species, often based on modeling and experimental researches (Cultura and Salameh, 2015; Veneri et al., 2018), such as carbon and silicon nanomaterials (Wan et al., 2021), natural clays (Lan et al., 2021), and metal–organic frameworks (Baumann et al., 2019), which have possessed some acceptable figures of merit like biodegradability, cost-effective, availability, and high-performance energy storage (Li Z. et al., 2013; Mitlin et al., 2013). These species often possess enormous efficiency compared with some artificial compounds (Someya et al., 2016). Although, with these species, no reliable symptoms are available in the literature in the point of view of their supercapacitive property (energy storage) (Someya et al., 2016; Vielreicher et al., 2018; Yang et al., 2019).

In addition, the term “*Parasite*,” which pointed to the “*Protozoans*,” “*Arthropods*,” and “*Helminths*,” inhabits some periods of its life cycle, in the body of another larger existent as the host (Mehlhorn and Mehlhorn, 2016; Bogitsh et al., 2018). Helminth eggs may contaminate soils, plants and even water sources (Slifko et al., 2000). Consequently, ingestion of helminth eggs through food and water would cause serious concerns and may cause parasitic infection and even death in humans and animals (Daszak et al., 2000; Chappell, 2013). However, from the initial point of view, these species are only known as a helminth, but they show significant characteristics, especially from the electronic aspect. The aim of this research is to provide a series of electrical probes to detect and differentiate between different types of helminth eggs. Also, due to the capacitive/resistance behaviors of the tested helminth eggs, they can be mentioned as a series of cost-effective bio-based supercapacitors. So, in this article, high supercapacitance, large power density, huge permittivity, large-scale (scalable) energy storage, as well as resistance (resistivity) of the helminth eggs are evaluated in detail. It seems that these characteristics can be adapted for direct detection and recognition of the helminth egg. More reliability, such as more precise analysis, fast and direct analysis time, no need to apply sample analysis, low cost, etc., seems to be needed compared with other traditional methodologies like direct visualization through optical microscopy (Suzuki et al., 2013), analysis by ELISA system (Mettler et al., 2005), etc. (Liance et al., 2000). These properties are attributed to the electrical/electronic behavior of the helminth egg, which are not only utilized as a detection probe but also noticeable for opening new horizons in the electronic technology in the near future. To the best of our knowledge, up to now, there are no introduced materials/compounds with completely mentioned energy storage/power density characteristics.

## Results and discussion

### Reagents/materials and instruments

All required reagents and solutions are reported entirely in the Supplementary Material (see the *Reagents and materials* section). The capacitance parameters of helminth eggs were determined based on the proper designs, which are fully described in the Supplementary Material (see the *Instruments* section). These parts completely covered all types of methods and instruments.

### Detection methodology

These designs are based on the “*Blind Patch-Clamp Methodology*” (Castañeda-Castellanos et al., 2006), at the “Giga ohm sealed condition” (Kornreich, 2007) via controlling the situation of the “*Implanted micro-electrodes*” on the eggshell of the helminth egg (Simeral et al., 2011). Their procedures are comprehensively explained in the Supplementary Material (see the *Procedure* section). About these descriptions, it is necessary to note that notations: “1,” “2,” “3,” “4,” “5,” as well as “6” refer to the types of helminth eggs, including “*Fasciola hepatica*,” “*Parascaris equorum*” (in the absence or presence of any larvae), “*Dicrocoelium dendriticum*,” “*Moniezia expansa*,” and “*Taenia multiceps*,” respectively.

The natural and intrinsic dielectric/resistivity behavior of the helminth egg is considered the fantastic point of view with tremendous solid-state supercapacitor performance. To reliably evaluate this inherent phenomenon, hereby in this report, the fundamental electrical aspects of the helminth eggs are assessed and discussed in detail.

### Selection of electroanalytical technique

According to our preliminary tests, which is stated in detail in the Supplementary Material (see *Part 1: unfeasible tests* section), to access the reliable capacitance value of each helminth egg, the electrochemical impedance spectroscopy (EIS) of each helminth egg was studied by using an electrochemical electroanalyzer as a programmable electrical waveform (function) generator, and data acquisition was performed by applying a single sinusoidal wave with frequency ranging between 0.1 Hz and 1.0 (± 0.1) MHz. To scan the potentials, the frequency range was divided into 50 sequential parts with logarithmic mode as the selected frequency step and the wave amplitude as large as 25.00 ± 0.01 (*n* = 3) mV (vs. total applied potential).

**FIGURE 1:**
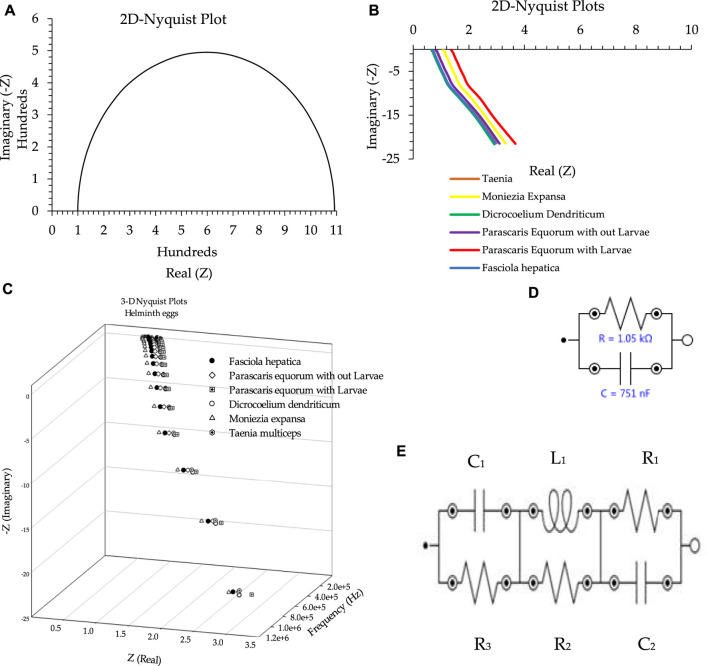
**(A)** Nyquist plot in the absence of the helminth egg. **(B)** Two-dimensional Nyquist plot for all types of helminth eggs. **(C)** Three-dimensional Nyquist plot as a sample of all types of helminth eggs. **(D)** Equivalent circuit as an example for the absence of any helminth egg. **(E)** Equivalent circuit as an example for the presence of all types of helminth eggs. Conditions: All conditions and procedures are expressed in detail as mentioned above during implanting two microelectrodes with 0.0124 ± 0.0008 mm inter-electrode distance, error bar: ± standard deviation (*n* = 5).

According to Figure 1A, in the absence of any helminth egg, the EIS showed only the characteristics of the external dummy cell, which was similar to the normal (RC) Nyquist plots (Figure 1D) (Abouzari et al., 2009). On the other hand, after setting blind patch-clamp connections at the giga ohm sealed condition, the imaginary part of the Nyquist plot (Figure 1B) showed negative values. This phenomenon, therefore, revealed very low capacitance impedance (X_c_), due to a huge capacitor (C), based on the X_c_ = 1/(j.C.ω) (ω = 2πυ, $\left(j=\sqrt{-1}\right)$ formula (Blatt and Blatt, 1989). These results were also besides accessing a very large inductance impedance (X_L_) because of a high inductor (L) as in the X_L_ = j.L.ω $\left(j=\sqrt{-1}\right)$ formula (Blatt and Blatt, 1989) and, consequently, magnetic permeability (µ) (Blatt and Blatt, 1989).

Based on the fitted equivalent circuit (Figure 1E), besides the presence of a common resistance, the existence of a considerable negative resistance was also proven for all types of the analyzed helminth eggs. To obtain the elements mentioned above, the electrochemical impedance spectra of all helminth eggs was analyzed entirely, and the results for all aspects are shown in Table 1.

**TABLE 1:** Electrical parameters related to the equivalent circuit.

Egg type	C_1_ (F)	L_1_ (H)	R_1_ (megaohm)	R_3_ (ohm) × 10^13^	R_2_ (ohm)	C_2_ (fF)
1	5.60	0.01	1.78	−0.72	2.455	0.045
2	11.37	0.05	2.95	−3.11	3.9144	0.184
3	16.67	0.09	2.66	−1.51	3.1932	0.192
4	2.28	0.003	1.22	−0.70	1.6167	0.023
5	2.20	0.001	2.37	−0.84	3.1212	0.028
6	15.90	0.08	3.02	−3.11	3.7128	0.215

Note. All conditions and procedures are expressed in detail as mentioned above during implanting two microelectrodes with 0.0124 ± 0.0008 mm inter-electrode distance, error bar: ± standard deviation.

This experiment resulted in an equivalent circuit with four independent key elements: capacitor, inductor, resistor, and negative resistor as mentioned . The existence of these electrical elements can enable us to be in a unique position to introduce a recognition probe for the testing and detection of helminth eggs. However, to simplify the results, only the general capacitor and positive resistor were thoroughly studied as novel criteria for the identification of helminth eggs.

### Supercapacitor behavior of helminth eggs

The supercapacitor behavior of each helminth egg was measured by three distinct procedures:

i) Direct capacity measurement using an analog multimeter (i.e., AVO meter) as a rough initial testing system,
ii) Indirect measurement by an integrating amplifier of an operational amplifier,
iii) Measuring the capacitance (i.e., capacity per area) based on the output electrical current density (based on the C = j.t/V formula) (Blatt and Blatt, 1989).

All the completed descriptions of the three methods are detailed in the Supplementary Material (see *Part 2: Capacitance* section).

**FIGURE 2:**
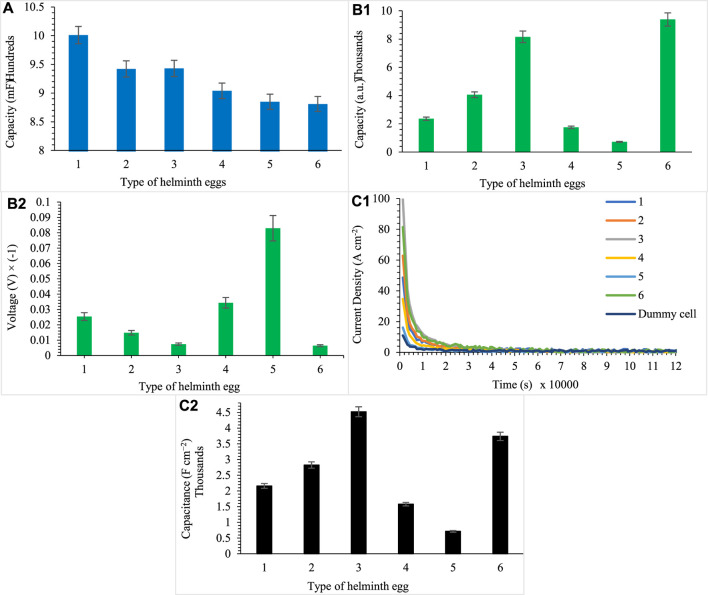
**(A)** Determination by multimeter, **(B)** determination by Op-Amp (integration) (b_1_, b_2_), and **(C)** current density of helminth eggs, capacity per area (c_1_, c_2_), for five independent repeats during implanting of three microelectrodes with 0.0124 ± 0.0008 mm inter-electrode distance, error bar: ± standard deviation (*n* = 5). Note that to prevent clutter, error bars are eliminated in part (c_1_).

Due to the obtained results from the first procedure (Figure 2A), it is concluded that each helminth egg has a substantial capacitance in its structure. So to verify these results, the following procedures were performed. The results of these methods also confirm the magnitude of the capacitance in the helminth egg structure. Also, these two procedures corroborated the results of each other, as shown in Figures 2B, C. According to this figure, each type of helminth egg has particular capacitance for differentiation and identification. For instance, by utilizing a simple electronic device (using a multimeter) to using complex instruments with the ability to measure capacitance, they can be enough to recognize different types of helminth eggs.

Due to reproducible supercapacitor behaviors of helminth eggs, this approach can be used to identify different types of helminth eggs. This behavior is almost attributed to the structure of the helminth eggs and is specific to each of them. Consequently, it hoped to differentiate these microorganisms with no need to use any operators and costly methods. In addition, these helminth eggs can be considered as a new window to the construction and inspiration of bio-based supercapacitors.

### Scan rate effect

One of the most important features of supercapacitors is their capacitance stability versus rapid voltage changes (high scan rate) or high current density (A cm^−2^ or A g^−1^) of the material that was directly analyzed. The capacitance stability of helminth eggs was evaluated via applying a constant (potentiostat) potential and measuring the flowing current and power. For these purposes, a constant potential was used on the working microelectrode (vs. the pseudo reference one) at OCP (open circuit potential) condition (Skoog et al., 2017). The OCP requirements were, therefore, provided at practically the zero-current condition (Martin, 2000) for access current as small as 1.71 ± 0.07 × 10^–11^ A (*n* = 5), using a voltage-follower circuit (Ramirez-Angulo et al., 2002). This circumstance, therefore, resulted in accessing the ideally non-polarized condition for the selected pseudo reference microelectrode, especially when dealing with a biological system such as a helminth egg as the selected sample with a high time constant (τ = R × C). At this condition, the DC potential (to reliably evaluate the helminth egg as a dielectric matrix) was scanned from −30.0 to +30.0 V (DC vs. GND, as the potential window of each tested helminth egg, without regard to any electrical damage and shock). The controlled current density was measured between the working and counter microelectrodes. However, due to the electrical damage (irreversible shocking) of the helminth egg at high current conditions, such as from 1.14 ± 0.0.9 × 10^–3^ A, *n* = 5), as the threshold electrical current, the level of the current was controlled by the potential-controlled patch-clamp technique via potentiometric measuring of the working electrode potential (vs. the pseudo reference micro-electrode by potentiometry). At this condition, the capacitance values were measured during the application of potentials with different scan rates, such as 1.0, 10.0, 20.0, 40.0, 60.0, 90.0, and 120.0 V s^−1^.

**FIGURE 3:**
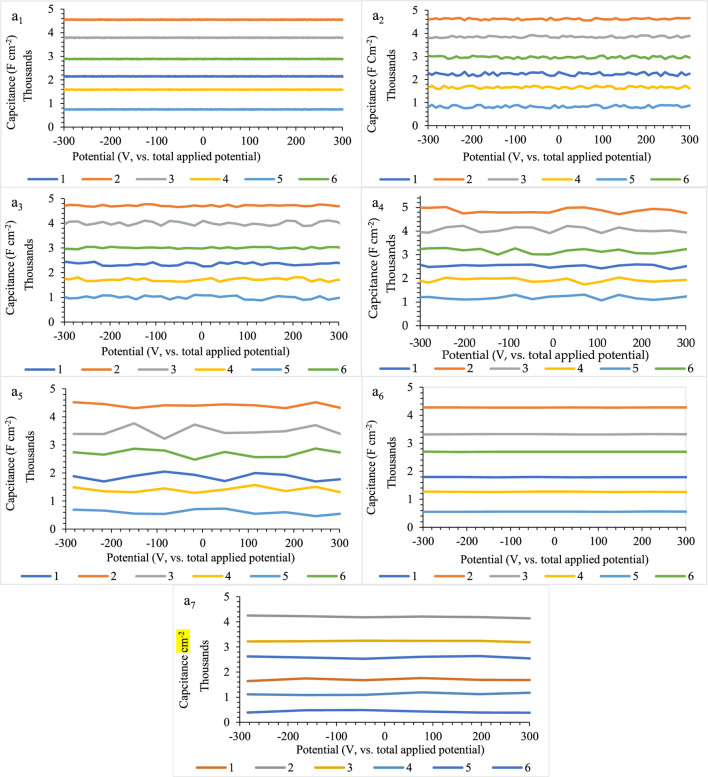
**(A)** Effect of scan rate on the capacitance stability (a_1_–a_7_) for five independent repeats during implanting of three microelectrodes with 0.0124 ± 0.0008 mm inter-electrode distance, error bar: ± standard deviation (*n* = 5). Note that to prevent clutter, error bars eliminated are from different parts of the figure.

However, at first glance, it seems that these tests do not have enough validation due to the lack of any current flow through the surface of the helminth eggs. Nevertheless, because of the significant time constant for the helminth eggs, which is due to the relatively great resistance and large capacitor, this approach is considered as the only way to investigate the effect of scan rate applied on helminth eggs. Therefore, due to the lack of significant changes in their capacity, it can be concluded that helminth eggs show excellent endurance to scan rates. Of course, this stability can be considered as the existence of different layers of water medium as a very suitable dielectric in the structure of the helminth egg. Figure 3A clearly shows the ability of helminth eggs to be stable in terms of scan rate.

According to the results of the scan rate, it can be concluded that using a simple device and without any worry of changing the capacitive behavior of helminth eggs at high speeds of the scan rate, a capacitive criterion as large as maximum can be utilized for recognition. So their capacitance results will be repeatable and reproducible after many evaluations.

**FIGURE 4:**
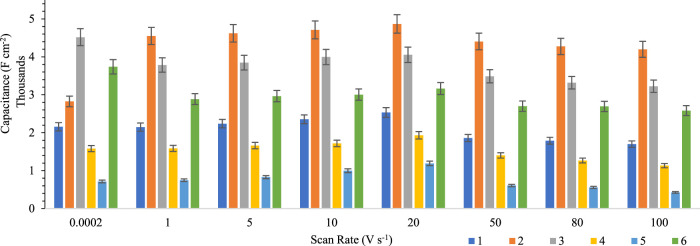
Comparison between the scan rate effects on the capacitance stability of helminth eggs for five independent repeats during implanting of three microelectrodes with 0.0124 ± 0.0008 mm interelectrode distance, error bar: ± standard deviation (*n* = 5).

In the end, for precise comparison of the potential scan rate effects on the helminth eggs, all the results are summarized in Figure 4.

Due to these results, the capacitance trend for helminth eggs is the same for different scan rates. Also, this trend for capacitance determination will lead to faster recognition of each helminth egg.

Owing to the results, it can be concluded that voltage changes have no significant effect on capacity fading. So, this feature can help in fast recognition without worrying about the changes in the capacity of the tested helminth eggs. In addition, the repeatable behavior of helminth egg capacity can inspire scientists to design a new generation of durable supercapacitors.

### Related capacity parameter

To better the capacitance description of helminth eggs, some features like capacity fading, energy density, power, and dielectric constant as intrinsic properties were also evaluated in detail.

To estimate the capacity fading, sequential (continuous) charging and discharging processes (at least 15,000 sequential cycles) were applied. For this purpose, each helminth egg was charged via applying a fixed step DC potential (to control the bipolarity of the helminth egg as a dielectric matrix) from 0.0 to +30.0 V vs. total applied potential (as the positive potential window of each tested helminth egg, without regard to any damage) at a fixed time interval between 1.0 and 20.0 s (as the response time for reaching to 100% steady-state condition, as well as a maximum time constant of the helminth egg charging/discharging process) to have complete confidence about completely charging the capacitance mode of each helminth egg. In addition, to completely discharge the capacitor of the helminth egg, before each test, the egg was connected to an external dummy cell for a maximum of 5.0 min (as the response time to reach maximum steady state condition).

**FIGURE 5:**
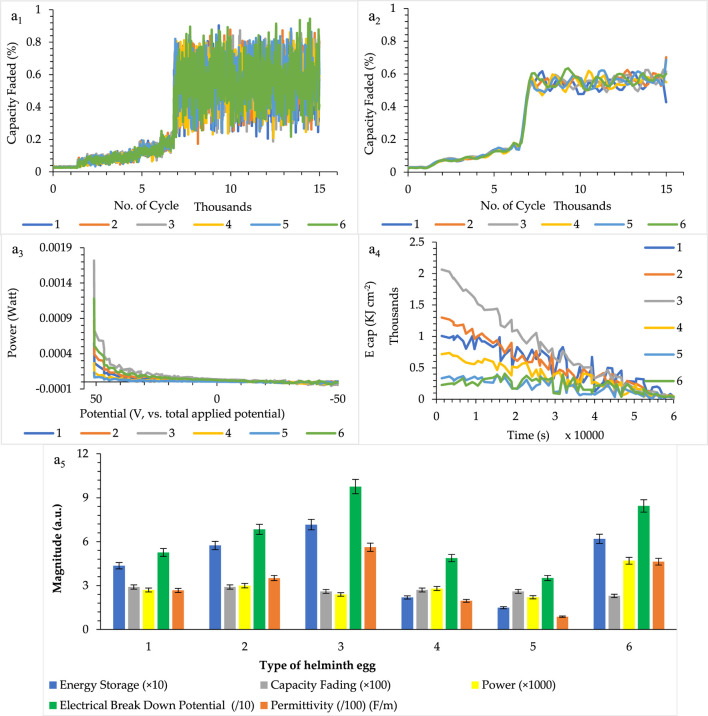
**(A)** Related capacity parameter [including capacity fading (nonsmoothed and smoothed), power, energy storage, permittivity, and electrical breakdown potential] for a single egg (a_1_–a_5_), for five independent repeats during implanting of three microelectrodes with 0.0124 ± 0.0008 mm interelectrode distance, error bar: ± standard deviation (*n* = 5). Note that to prevent clutter, error bars are eliminated in parts (a_1_–a_4_).

As can be seen in Figure 5, helminth eggs lose only 1% of their capacity in high numbers of cycles due to structural stability under high applied cycle voltage. It seems that the structure of the helminth egg would revert to its initial structure after each cycle. It can, therefore, be concluded that the electrical charging–discharging phenomena are reversibly processed that results from the layer-by-layer structure of water and biological components in the helminth eggs.

In addition to this parameter, other vital aspects of the helminth eggs, such as electrical power, energy density, and the electrical breakdown potential, were also considered. As can be seen in Figure 5, these creations have high power per egg as well as a high energy surface density, which, in addition to high breakdown voltages, are considered as the amazing phenomenon in the field of solid-state supercapacitors. These fantastic features are shown in Figure 5 ultimately.

To evaluate the permittivity (dielectric constant) of each type of helminth egg as an intrinsic aspect, according to the formula C = ε × A / d (Eq. 2), the magnitude of permittivity (F m^−1^) of each tested helminth egg was measured by the surface area of each helminth egg as “*A*” notation, which is explained in the Supplementary Material (see *Part: Surface area-average surface area of the helminth egg* section), and the inter-micro-electrode distance as “*d*” notation, which is expressed in the Supplementary Material (see *Part: Surface area-helminth eggs inter-microelectrode distance*). This condition, therefore, resulted in measuring the capacitance value per the surface area of the helminth egg based on the third method of capacity measurement. The results, as well as their comprehensive data, are shown in Figure 5.

Due to the extraordinary durability of helminth eggs in the electrical charge/discharge processes, these creations were made as long lifetime supercapacitor candidates. However, on the other hand, large energy density and high power density per each tested egg caused these natural biomaterials to play a role as a suitable electrical energy storage. In addition, high electrical breakdown voltage is another amazing feature (aspect) for the helminth eggs. Besides these fascinating aspects, huge electrical permittivity can, therefore, be proven. All these results and conclusions may be related to the existence of nanodielectrics, which were constructed by water adsorption in the biological component of the helminth eggs. According to the different parts of Figure 5, helminth eggs can, therefore, be a novel and suitable material in the energy storage field.

In addition to the figures of merits mentioned above, using these parameters as an indicator (detection probe) to distinguish the types of helminth eggs from each other can also be of use. Due to the high durability of the helminth egg, this method can be registered as a direct, fast, simple, and non-invasive recognition method. Especially, the existence of high electrical breakdown voltage can be a rapid, accessible parameter for tested helminth egg identification. Also, the electrical permittivity of each helminth egg is the critical parameter for proving these differences. Therefore, all mentioned parameters can be used with acceptable repeatability and reliability for detection and recognition purposes. These features can be attributed to the construction of the helminth eggs and their components. It seemed that increasing the structural order of water molecules led to more capacity and its related parameters such as in *Parascaris equorum* larvae. So, the capacity behaviors of helminth eggs are related to their unique structure.

So, it can be concluded from capacitance measurements that the capacity of each helminth egg can be used as a criterion for differentiation between different types of helminth eggs. In addition to this feature, several related parameters such as energy density, power density, and electrical breakdown voltage can be utilized for recognition of the helminth eggs. Also, electrical permittivity as an intrinsic feature of capacity can be employed for the helminth egg detection purpose.

Also, the distance between electrodes was fixed due to the elimination of any changes to the evaluated parameters, such as capacity, resistance, energy density, power density, and other related parameters. It also should be noted that due to the dealing with the giga ohm sealed condition, as well as the same microelectrode system, the same peak area (A) was chosen for evaluation of the capacity values. Also, based on the C = ε × A / d, the minimum possible electrode distance (20 μm) was selected for access to maximum possible capacitance, experimentally. Along with this, the size of *Taenia multiceps* helminth egg with a maximum length of 37 μm (Urquhart et al., 1996) limited the test of more electrode distances. Nevertheless, as we deal with the same (A / d) values for all the tested helminth eggs, the electrode distance caused no problem(s) during the comparison between the capacitance values of different helminth eggs. So, there was no need to optimize the interelectrode distance.

### Helminth egg resistance measurement

The resistance of each helminth egg was measured by two procedures:

i) Direct resistance measurement by using the LCR meter,
ii) Estimation of the resistance by linear sweep scanning of a potentiostat voltage from −300.0 to +300.0 mV (vs. total applied potential) and measuring current according to Ohm’s law. As an appropriative feature of the helminth egg structure, the related resistivity (ρ, Ω cm) was consequently measured similar to what was mentioned above for the dielectric constant measurement.

**FIGURE 6:**
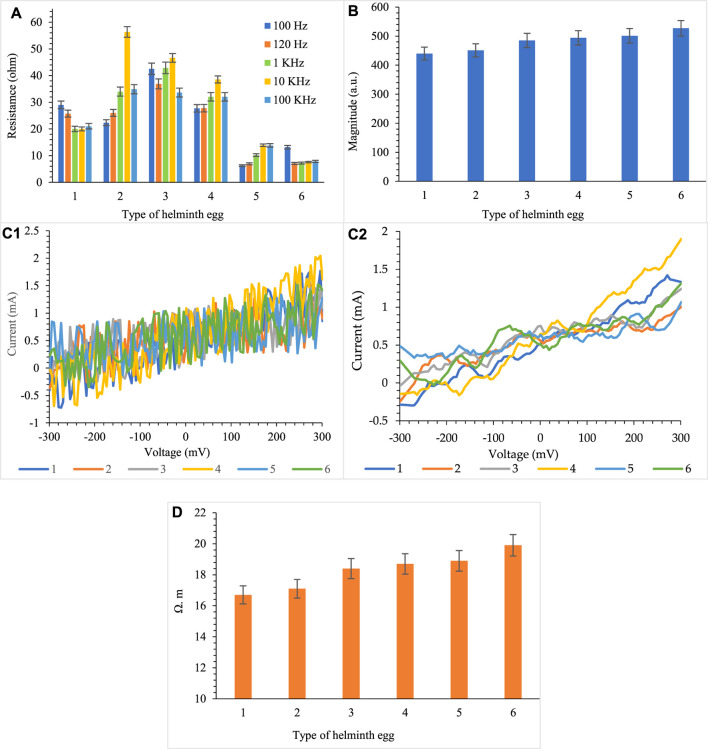
The resistance determination by **(A)** LCR meter, **(B)** DC resistance, **(C)** non-smoothed and smoothed normal DC resistance of helminth eggs (c_1_, c_2_), and the **(D)** resistivity measurement, for five repetitions during implanting of three-microelectrodes with 0.0124 ± 0.0008 mm inter-electrode distance, error bar: ±standard deviation (*n* = 5).

Based on the results, the helminth eggs, in addition to having a huge capacitor in their structure, also have a relatively high amount of electrical resistance. Due to the initial experimental results (which was considered as a preliminary method by using the LCR meter), the existence of electrical resistance in the helminth eggs can be confirmed (Figure 6A). To validate this result, a subsequent experiment was performed to demonstrate this feature fully. It can be concluded from the experiments that due to the large area of the surface layer of helminth eggs and the presence of water as a discrete medium [during its synergistic effect(s) probably with other biological materials in the structure of the helminth egg], they also show resistance behavior.

In addition to the above-reported diagnostic indices based on the capacitive properties of helminth eggs, the use of resistance (electrical resistivity) indices is also an effective solution in diagnosing different types of helminth eggs. It seemed that the polymeric construction of the membrane and other biological components of the egg played a vital role in resistance behavior.

However, the resistance behavior of the helminth egg was not so acceptable in comparison with the capacitance behavior, but the resistance behavior of eggs can also be a good indicator of diagnosis.

As clearly shown based on the multiple analyses of different helminth eggs under similar conditions, fortunately, no significant systematic errors (bias) are observed in the results (Figures 2-5). In addition, the amount of estimated %RSD (5%, Figure 5) pointed to the acceptable reproducibility of this introduced biosystem for both capacity applications and detection purposes. These pieces of evidence revealed this fact, despite the presence of various natural physiological and environmental differences in the natural biosystems, such as the helminth egg. Still, these functions did not provide any significant problems(s) in these analysis processes. This observation was probably attributed to the different aspects, especially, i) the capacitance behavior of the helminth egg at the cellular levels, and ii) the experimental method and the selected procedure adopted for this process.

### Semi-qualitative proposed interpretation (a proposed mechanism)

According to what the researchers reported so far, biological compounds, such as proteins (Velásquez et al., 2004), lipids (Sasz and Lescure, 1966), phenolic compounds (Schiller et al., 1975), carbohydrates (Passey and Fairbairn, 1957), water (Maule and Marks, 2006), and oxygen (Barrett, 1981), are often present in the structure of the helminth eggs. However, exact amounts, sequence, and configuration of these biological units in the matrix of the helminth egg have not yet been reported precisely. Although the structures of helminth eggs are similar to each other in many cases, such as the existence of proteins, lipids, phenolic compounds, carbohydrates, as well as water and oxygen, they have different configurations in their structures, according to literature.

In parasitic nematodes, such as Parascaris equorum, the membrane of the egg contains an inner lipid layer that is different from the other helminth eggs. It should be mentioned that their membranes can have different thicknesses of various components, such as lipids, chitin fibrils embedded in a protein matrix. Whereas lipid layers are less common in parasitic cestodes and trematodes. The egg shell of many trematodes is sclerotin (a quinone-tanned protein) that may be a form of collagen with a high content of tyrosine. Beneath the egg capsule of cyclophyllid cestodes, there is a granular gelatinous layer. However, in taeniid cestodes the greatly thickened embryophore is not sclerotin and may be a type of keratin. Nevertheless, only a few reports about the helminth egg formation has been reported (Smyth and Clegg, 1959; Wharton, 1980). Regardless, to understand the role of the helminth egg materials in the parameters mentioned above, the helminth eggs were precisely weighted once without/with heating up to 60.0 ± 1.0°C in the vacuum oven at 720.0 ± 0.3 Torr pressure with a 5.0-h time interval.

According to Table 2, the weight ratio between the removed and the remaining material was in good agreement with the proposed behavior. After applying the heating procedure, all the tests and calculations were again repeated (under similar conditions) to evaluate the effect(s) of the removed materials. As shown in Table 2 (two columns from the right), all the reported parameters were experimentally minimal. This result, therefore, pointed to the fact that each egg acted as a non-repeatable resistor.

**TABLE 2:** Effect of lost weight on the electrical parameter.

Type of helminth egg	Fresh average weight ± SD (*n* = 10) (g)	Dried average weight ± SD (*n* = 10) (g)	Removed average weight/fresh average weight ± SD (*n* = 10)	Capacitor quantity after drying (F/cm^2^) ± SD (*n* = 10)	Resistance quantity after drying (Ω) ± SD (*n* = 10)
Fasciola hepatica	0.0034 ± 0.0028	0.0018 ± 0.0015	0.44 ± 0.00	0.37 ± 0.42	48.12 ± 7.36
*Parascaris equorum* without larvae	0.0054 ± 0.0027	0.0049 ± 0.0024	0.09 ± 0.00	0.91 ± 0.38	76.05 ± 13.81
*Parascaris equorum* with larvae	0.0038 ± 0.0025	0.0027 ± 0.0018	0.28 ± 0.00	0.46 ± 0.24	54.27 ± 17.15
*Dicrocoelium dendriticum*	0.0031 ± 0.0023	0.0014 ± 0.0004	0.52 ± 0.00	0.07 ± 0.04	34.61 ± 23.08
*Moniezia expansa*	0.0058 ± 0.0011	0.0024 ± 0.0004	0.53 ± 0.00	0.42 ± 0.31	48.13 ± 27.14
*Taenia multiceps*	0.0054 ± 0.0029	0.0023 ± 0.012	0.56 ± 0.00	0.36 ± 0.27	71.42 ± 15.11

Note. The results were reported based on five repetitions during implanting of three-microelectrodes with 0.0124 ± 0.0008 mm interelectrode distance, error bar: ±standard deviation.

The materials (especially water molecules along with the dissolved O_2_ and CO_2_) that are generally released from the body of the egg played a significant role during the appearance of these intrinsic behaviors. In addition, based on the reported research articles (King et al., 1991; Bibi et al., 2016), the dielectric constant of protein changed from 0.0 to 80.0 arbitrary units (a.u.), which is following the quantity of the absorbed water medium. This is based on their three-dimensional configurations and the spherical isomerization of the amino acid matrix (Dwyer et al., 2000). It appears that orientation and configuration of different components, especially the proteins that made up the 3D distributed vitelline cells, are considered as an independent dielectric (Dwyer et al., 2000) in different series and parallel modes (Halliday et al., 2003). Therefore, they create a very high capacitance while absorbing the water medium. Thus, the helminth eggs showed an enormous capacity.

In accordance with Table 2, evidently, more amounts of water media in the structure led to more significant irregularity in the helminth egg medium. This process mainly reduced the capacitance values, as can be seen in *Dicrocoelium dendriticum*, *Moniezia expansa*, and *Fasciola hepatica*. Consequently, lowering the amounts of water medium in the larvae structure directly resulted in manipulation of the level of orientation of the water molecules toward these proteins (Dwyer et al., 2000). Therefore, by decreasing the amount of water, helminth eggs act as a relatively ideal dielectric material as can be observed in *Parascaris equorum* (with and without larvae) and *Taenia multiceps.* This phenomenon may be related to lowering water contact between each neighboring vitelline cells. However, to further evaluate the influential roles of the water medium on the capacity performance of the helminth eggs, two procedures were applied to these eggs:

The first one is based on humidifying via soaking a previously dried egg (according to the recommended procedure) inside a water medium (100.0 ml). This process was achieved at different temperatures (ranging between 25°C and 50°C) and at both dark and light conditions. In addition, the other step was related to the presence of some ionic species, such as NaCl solution with 0.10 mol L^−1^ concentrations for at least > 10.0 h.

Also, based on the second procedure, the helminth eggs were individually situated inside a closed bottle (glass balloon, 50.0 ml), containing water aerosol with a relative humidity (RH) of >60% generated using a humidifier system for a long time (>10.0 h).

In conclusion, the lack of appearance of any capacity behavior on the helminth egg even after applying at least one step of the drying procedure to each helminth egg powerfully revealed the effective role(s) of the water medium as well as the significant (irreversible) influence(s) of the desorbed water during the denaturation of the helminth eggs. In addition to the heating mentioned above, ethanol was also used to inactivate the helminth eggs. The experiments were repeated, with the result being the same as the heating mode.

## Conclusion

In this study, for the first time, a novel probe for rapid identification and direct differentiation of helminth eggs has been introduced based on their different supercapacitance/resistance behaviors. However, the presence of an electrical layer with a negative excess electrical surface charge has been previously proven based on the literature (Capizzi and Schwartzbrod, 2001). Still, this study, besides the introduction of biological energy storage bank, strongly exhibited the possible significant performance of natural species in the development in all parts of science in the near future. To the best of our knowledge, this study can be regarded as the first report in which the helminth eggs can be considered as a natural solid-state supercapacitor. For better presentation of the advantages of the helminth egg as a nature-inspired (mimic) supercapacitor device, we compare their particular parameters like capacity, capacity fading (retention), and power density by some other investigation in Table 3. It can be concluded that, even the inferior type of helminth egg is more robust than other previously introduced supercapacitor-based materials in different attitudes.

**TABLE 3:** Comparison between this work and some other published reports.

Materials used in supercapacitor	Type of super capacitor	Capacity in different scale (F g^−1^) or (F cm^−2^)	Capacity fading in different scan rate	Power density	Reference
Helminth egg (Present Work)	Solid-state	714.0–4,519.0 F cm^−2^	1% after 15,000 cycles in different scan rates (2.0 × 10^−4^–120.0 V/s)	(2.2–4.7 W/egg)	-
PEDOT: PSS/MnO_2_ nanorods/RGO nanocomposite	Hybrid	633 F g^−1^	10% after 5,000 cycles in 1 A/g	-	Hareesh et al. (2017)
Amorphous nickel hydroxide nanospheres [Ni (OH)_2_]	Electrochemical	2,188 F g^−1^	(97 and 81% charge retentions after 5,000 and 10,000 cycles)	490 W kg^−1^	Li et al. (2013a)
Coaxial wet-spun yarn	Electrochemical	177 mF cm^−2^	75% at a high current density of 1 mA cm^−2^	3.84 mWh cm^−2^	Kou et al. (2014)
Mesoporous nitrogen-rich carbons	Electrochemical	390 F g^−1^	7% fading after 10,000 cycles	-	Li et al. (2013b)
Micrometer-sized onion-like carbon	Electrochemical	0.4–2 mF cm^−2^	-	1 × 10^–2^ Wh cm^−3^	Pech et al. (2010)
Macro-porous Ti_3_c_2_T_*x*_ MXene	Pseudo-capacitor	210 F g^−1^	80% after 10,000 no. of cycles in 1 A g^−1^	-	Lukatskaya et al. (2017)
Fractal (Ni_*x*_Co_1-*x*_)_9_Se_8_ nano dendrite	Solid-state	3,762 F g^−1^	94.8% of the initial capacitance after 5,000 cycles	1 × 10^4^ W kg^−1^	Yang et al. (2018)

As shown in detail in Table 3, these characteristics can be adopted for direct detection and recognition of the helminth egg. It seemed that these creatures could compete with other types of supercapacitors, such as electrochemical, pseudocapacitor, hybrid, and solid state in their ability of energy storage. In addition, the abundance, low price, biodegradability, and of course, the high durability of these microorganisms prove their competitiveness compared with other synthetic materials. On the other hand, the supercapacitor behavior of helminth eggs can be considered as a combination of different behaviors of other conventional supercapacitors due to their structures. In the future, these creatures could open a new window into the needlessness of building supercapacitors. Higher advantages, such as more precise analysis, fast and direct analysis time, no need to apply sample analysis, low cost, etc., seem to be required compared with other traditional methodologies like direct visualization through optical microscopy or analysis by ELISA system, etc. These properties, therefore, attributed to the electrical/electronic electrical behavior of the helminth egg, cannot only be utilized as a detection probe but also can be noticeable for opening new horizons in the electronic technology in the near future.

## Data Availability

The original contributions presented in the study are included in the article/Supplementary Material. Further inquiries can be directed to the corresponding author.
