# Supplementary material for: Supercapacitance/Resistance Behaviors of Helminth Eggs as Reliable Recognition and Direct Differentiation Probe
Source: Front Bioeng Biotechnol. 2021 Dec 6;9:782380. doi: 10.3389/fbioe.2021.782380 (PMC8685256; doi:10.3389/fbioe.2021.782380)
Supplement: Supplementary file 1 [file DataSheet1.docx]

***Supplementary Information***

**Related to**

**Supercapacitance/resistance behaviors of helminth eggs as novel horizons for both energy sources and differentiation probe**

Ruhollah Shaali^1^, Mohammad Mahdi Doroodmand^1,*^, Mohammad Moazeni^2^

^1^ Department of Chemistry, Shiraz University, Shiraz, Iran

^2^ Physiological division of Department of basic science, School of Veterinary Medicine, Shiraz University, Shiraz, Iran

*Correspondence: [doroodmand@shirazu.ac.ir](mailto:doroodmand@shirazu.ac.ir), doroodmand@yahoo.com (M. M. Doroodmand), Tel: +98-71-36137152, Fax: +98-71-36460788

**Reagents and materials**

All the reagents were purchased from their analytical grades. Solution of 1.00 ± 0.01 % (W/V) of NaCl (99.9 %, Merck Company) was prepared by deionized water (DIW or de-ionized water, specific resistivity: of 17.5 ± 0.2 MΩ cm, Direct-Q® Water Purification System, France). HAuCl_4_ (>99.9 %, Merck Company) as the Au nanoparticles precursor and ascorbic acid (Analytical grade, >99.0 %, Merck Company) as both the reducing and capping agents were utilized to generate the Au nanoparticles. Ultra-analytical grade of ethanol (C_2_H_5_OH, 99.0 %, Merck Company) was used to kill (destroy) the helminths eggs.

**Instruments**

The adopted instrumentation systems included:

-A cubic faraday’s cage: (Dimension: 50.0 × 50.0 × 60.0 cm, stainless steel, Type: 308),

-Voltmeter meter (Fluke 1507 Digital Megohmmeter Insulation Resistance Tester, USA),

-Ohmmeters with different models including Ohmmeter (Fluke 179 True RMS Digital Multimeter with TPAK toolpak magnetic meter hanger kit, USA), Ohmmeter (10.0 kV megger insulation resistance tester, KD2677D, Accuracy: ±2 %, Japan), Resistance tester (Fluke 1507 Digital Megohmmeter Insulation Resistance Tester, USA), and Ohmmeter (Digital Multimeter, Fluke 115 True RMS, USA),

-Electroanalyzer (Autolab, ECI10M, frequency range: 10.0 MHz to 100.0 (±0.1) µHz Input impedance: 100.0 GΩ, Metrohm AG, Ionenstrasse, CH-9100 Herisau, Switzerland),

-Autoclave (NanBei, Qingdao, China),

-“Stainless Steel Microelectrode” (Epoxy-insulated stainless-steel microelectrode, type: 308, diameter: 0.010 in., length: 3 in., tapered tip size: 12 °, A-M Systems^TM^, 131 Business Park Loop Sequim, WA 98382, U.S.A., Catalog #: 572700),

-Inverted fluorescence microscope (Trinocular head; Top video port, Germany),

-High-speed Charge Coupled Device, (CCD camera system (Full HD, 1000X (Manual), USB Adjustable Digital Microscope Camera Magnifier with 8 LED, light-emitting diode, illuminator, Picture Resolution: 1960×1080, Video Capture Resolution: 0.3-2.0MP, Frame Rate: 30 f/s under 600 LUX Brightness, Model Number: S1000 X, Guangdong, China), (Elmo 9711X3 model TNC4614X High-Resolution CCD camera Color, Samsung, Assembled in Japan),

-“High Resolution-Atomic Force Microscope*”* (HR-AFM, Tweezers (LANKIZ Eyelash Extension Tweezers, Stainless, Qingdao, China),

-“High Resolution Transmission Electron Microscopy” HR-TEM, (TEM FEI Tecnai G2 20 X-Twin, 200 kV, USA),

-HR-AFM, (Vibrating, Non-Vibrating, Phase, Lateral Force, Zoom to 400X, 2µ resolution, LabVIEW™ software, UK),

-Transmission Electron Microscopy (TEM, JEOL ARM300F GrandARM TEM, CEA, Saclay, France),

-Operational amplifier: (HMC1001, Operational Amplifier operational Summing Amplifier, LTC1150CN8#PBF Analog Device) according to *V_out_=-{R_f+_R_egg_}/R_i_) × V_in_* formula, where *R_f_*: feedback resistor, 10.000 (±0.001) KΩ, *R_i_*: input resistor, 100.000 ± 0.001 KΩ, V_in_: input potential, Military Series (*RLR*) metal film resistors, MIL-PRF-26, USA),

-Capacitive alternating voltage divider (Werner Dr Ing Strauss, DE19648230A1, 1996, Germany),

-Dummy cell (Autolab, RC circuit, R: 1 KΩ, C: 1 µF, Metrohm^AG^ Company),

-Glue film (2×2 cm, Zhejiang Shichuang Optics Film Manufacturing Co., Ltd., China), Glue film (Vistamaxx Performance Polymers, High Elastic TPU Hot Melt Adhesive Film Thermo, 1.75 ± 0.05 mm, Shenzhen Tunsing Plastic Products Co., Ltd., China),

-Glue paste (Vistamaxx Performance Polymers, High Elastic TPU Hot Melt Adhesive Film Thermo, 1.75 ± 0.05 mm, Shenzhen Tunsing Plastic Products Co., Ltd., China),

-3-dimensional printer (Design via modification of the three-dimensional printer, Pxmalion, B07DYN48FZ, PX-MINI, Accuracy: (X, Y, and Z dimension): 0.01 mm, Position accuracy: 0.01 mm, dimension: 30×30×20 cm, resolution: ± 0.01 mm, LulzBot TAZ 6, USA),

-Digital Micrometer (Mitutoyo 395-353 - MIC, DIG SPH FACE, 3"/76.2MM, Spherical Face Micrometer, Series 395 Digital model with a spherical anvil, US),

-High-capacitance meters (Capacitance meter, 0.001fF - 15 mF | 3505, 3506, HIOKI, English),

-LCR meter (GW Instek, 900 Series Handheld USA),

-Capacitor (183 µF, 5.0 V, Military MIL-PRF-55365/8, USA), Capacitor: (10.0 µF, 100.0 V. Military Series MIL-PRF-55365/8, USA),

-Resistance (R=1.000 Ω, Military Series (*RLR*) metal film resistors, MIL-PRF-26, USA), Resistor: (R=1.000 KΩ) Military Series (RLR) metal film resistors, MIL-PRF-26, USA),

-BET specific surface area analyzer (V-Sorb 2800S, China(,

-Micro stepper motor (Size: 42.3 mm square × 48 mm, CHANGZHOU JKONGMOTOR CO., LTD., China),

-Coaxial wires (RG6, CIMPLE CO - 30' Feet, USA),

-BNC, connector, China),

-FE-SEM (JSM-7500F Field Emission Scanning Electron Microscope, JEOL, Japan),

-High- Resolution TEM (*HR-TEM,* TEM FEI TecnaiG2 20 X-Twin, 200 kV, USA),

-Potentiometer (Multi-turn potentiometer: 100.00 KΩ, military Series, China),

-Mechanical interfaces (Lead Screw, length 10.0 cm, Bipolar, 400 Steps/Rev, Nema 11, China),

-Ultra high-resolution gravimeter (Analytical Balance, AE 240, Mettler, USA).

**Collection of helminth eggs**

*Parascaris equorum* eggs were obtained from the specimens, referred from the animal clinic, School of Veterinary Medicine, Shiraz University, Shiraz, Iran. In addition, *Fasciola hepatica* and *Dicrocoelium dendriticum* eggs were collected from the adult worms, obtained from the livers of naturally infected sheep slaughtered at Shiraz slaughterhouse (Zarghan, Fars, Iran). The *Moniezia expansa* eggs were obtained from the gravid proglottids of adult worms collected from the small intestine of naturally infected sheep slaughtered at the Shiraz Slaughterhouse. *Taenia multiceps* eggs were obtained from the gravid proglottids of adult worms collected from a naturally infected dog referred to the small animal clinic of School of Veterinary Medicine, Shiraz University (Shiraz, Iran).

The uterus of adult female *Parascaris equorum* and uterine area of adult *Fasciola hepatica* and *Dicrocoelium dendriticum* and gravid proglottids of *Taenia multiceps* and *Moniezia expansa* were separately crushed by a mortar and pestle, dissolved in unchlorinated water, and passed through a 500-µm mesh sieve to separate coarse tissue residues from the eggs. Subsequently, the passed materials, containing the eggs were washed several times with unchlorinated water in 50.0, 25.0, and finally 10.0 mL calibrated cylinders for 20.0, 15.0, and 15.0 min, respectively. After the total removal of the supernatant, the sedimented eggs were transferred into 2.0-mL microtubes, containing phosphate buffer solution (PBS, 1X) and stored at 4.0 ± 0.5 °C until use.

**Technique**

High-frequency impedance spectroscopy included Autolab system (ECI10M, frequency range: 10.0 MHz to 100.0 (±0.1) µHz, **Input impedance**: 100.0 ± 0.1 GΩ, Metrohm AG, Ionenstrasse, CH-9100 Herisau, Switzerland).

**Procedure**

In order to design an experiment for the determination of the capacitance and resistance of all the eggs, by multi-meter, capacitometer, and resistometer, LCR meter, and the *EIS*, the below instruction was adopted. Briefly, the adhesive glue film was connected to the two horizontal directions (i.e. *X and Y* axes) plane of the three-dimensional printer. After that, the selected helminth egg was immobilized and fixed on the surface of the glue film (paste). The direct electrical connection to the support as the electrode system was also set via controlling the size of the adhesive glue. After proper waiting time for drying (1.0 h per each mL of the fluid), the analysis process was operated. The Giga ohm sealed condition was then provided according to the suggested procedure. After that, all the capacity parameters were measured by the recommended procedures. The optimization is based on the one-at-a time method.

**Blind patch-clamp methodology**

To estimate the capacity behavior of each helminth egg, the three-microelectrode system including working, pseudo reference, and counter probes was implanted onto the helminth egg’s shells with 0.0124 ± 0.0008 mm inter-electrode distance (See part Surface area- helminth eggs inter-microelectrode distance). For this purpose, the X-Y positions of each egg were set using the X- and Y-axes of the 3-D printer. Whereas, the three-microelectrode system was connected to the Z-axis with a triangular shape and inter-electrode distance of 0.0124 ± 0.0008 mm using three independent stepper motors (Full cash) and mechanical interfaces, linearly connected to each electrode system, independently. A constant DC/AC potential (voltammetry) was applied to the working electrode versus the pseudo reference microelectrode, along with potentiostat electrical parameters measurement on the surface of the working microelectrode versus the counter one. It should be noted that “Blind Patch-Clamp” was adopted in this work, because of unclear and randomized electrical signal acquisition from the helminth eggs.

**Helminth egg’s Giga ohm sealed condition**

The Giga ohm sealed condition (G*Ω cm^-1^*) of each helminth egg was provided prior to the blind patch-clamp process via two independent procedures:

1. Direct measurement using a resistance tester connected the working and counter microelectrode system with 0.0124 ± 0.0008 mm inter-electrode system as the ohmmeter probes ^1^
2. Estimation of the output potential (*V_out_*) of an op-amp (operational amplifier) based on utilizing an adder circuit of an operational amplifier during introducing a 100.00-mV (DC, vs. GND) potential with small enough noise shunt-mode reference input potential, as a reference potential generator. This system was serried with the helminth egg as the feedback resistor. The output potential was measured using an oscilloscope that admitted the presence of a linear correlation between the current and applied voltage axes, based on the ohm’s law. At this condition, the resistance wave can be measured using the slope of the current-voltage (*I-V*) curve ^2^.

**Ohmmeter at zero current condition for the Giga ohm sealed conditions estimation**

The Giga ohm sealed condition was provided based on a reported procedure ^3^, briefly, via i) Blind controlling the forward and backward positions of the microelectrodes, linearly, using the stepper motors, implanted on each helminth egg, along with direct measuring the resistance using a digital ohmmeter. At this ohmic condition, obviously, besides the faraday’s cage, each helminth egg was conditioned at 6.18 ± 0.12 GΩ/cm (n=5) for data recognition sampling from each helminth egg at the provided high resistivity (impedance) conditions. This process majorly filtered most of the external random perturbations during the direct potentiometric sensing of the egg’s activities using the two-microelectrode system.

**Micro-electrode implant**

To investigate the capacity behavior of the helminths eggs, the microelectrode system was directly implanted for the blind patch-clamp method as the selected detection system. Then, three-microelectrodes were also implanted on the surface of the helminth egg from different situations using mechanical interfaces and the *Z*-cantilevered arm of the 3-D printer with the inter-electrode distance of 0.0124 ± 0.0008 mm. This process led to detecting the microtip position using the CCD camera as well as the simultaneous measuring of the helminth egg cell electrical resistance (Ω cm^-1^ to reach the Giga ohm sealed condition).

**Results**

**Part 1**: ***Unfeasible tests***

According to the capacity measurement by the multi-meter, it was concluded that each type of the tested helminth egg processed a very large capacitance (> 0.10 F per each tested egg), which was not quantitatively measurable by high-capacitometers, because of access to the rapid overload condition. However, a rapid change in the capacitance ramp, versus the time of an analog capacitance meter with an average velocity of 1.00 ± 0.01 ^o^ per section, was followed by the high-speed CCD camera. This scanning qualitatively confirmed the high capacitance of each tested helminth egg. This phenomenon was further evidenced via using high-frequency impedance spectroscopy, during direct estimation of the equivalent elements such as resistance and capacitance by the two-microelectrodes system (during using the copper plate as both supports, besides connection of the and pseudo reference plate and a microelectrode as working (by NOVA 1.6 software). However, the lack of observation of any reliable electrical response(s) probably pointed to the intrinsic behavior of the helminth eggs. Consequently, it was decided to divide the capacitance (1000.0-fold excess) using an external capacitive AC voltage divider circuit, set up as both an external dummy cell and the current filter. This process was especially operated in the AC mode for prevention from any break-down condition as well as the possibility to apply a fixed potential (+25.0 mV, vs. total applied potential) (Figure S1).


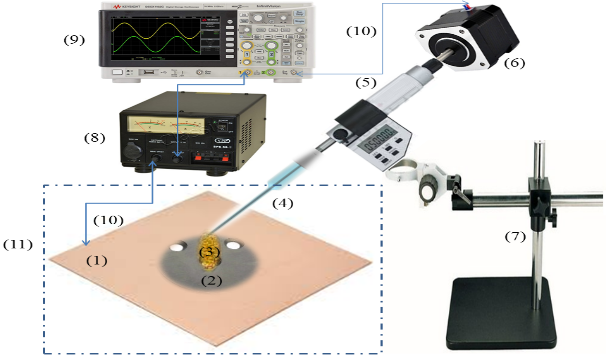


5.0 cm

**Figure S1. |** Schematic of the dummy cell designed to estimate the capacitance of the helminth egg. 1) copper plate, 2) sample holder well (diameter: 2.00 mm, height: 1.50 mm), 3) helminth egg, 4) microelectrode, 5) mechanical interface (digital micrometer), 6) stepper motor and driver, 7) base, 8) AC switching power supply, equipped with capacitive AC voltage divider circuit dummy cell, 9) oscilloscope, 10) coaxial wires, and 11) faraday’s cage. The analysis was achieved at standard temperature and pressure (*STP*) conditions.

**Part 2: *Capacitance***

The capacitance of each helminth egg was measured by three procedures:

-i) Direct measurement using a capacitance mode of the multi-meter using an external capacitor (1000.0 µF) that serially arranged with the helminth eggs to have capacitor-capacitor circuit as a preliminary test,

-ii) Estimation of output current density and consequently the capacitance cm^-2^, versus time during scanning of potential from certain voltage limits between ± 30.0 V (DC, vs. the ground, GND) at a fixed linear sweep scan rates (0.200 ± 0.001 mV s^−1^). At these conditions, the charging/discharging time constant (i.e. 63.2 % of charge or discharge) ^4^ of each helminth egg had majorly been higher than the scan rate time constant, to have full confidence about the reliability of the current density as well as the capacitance cm^-2^ versus time. Based on this hypothesis, the electrical energy stored at each egg as capacitance can be estimated using the E_cap_ =1/2 CV^2^ formula (Eq. 1) ^5^, at where E_cap_ is the energy stored in each egg as a capacitor (*C*) during applying a potential (V, vs. the GND).

-iii) And finally, estimation of the output potential (*V_out_*) of an integrator amplifier (op-amp integrator) ^6^ during introducing a 1.000-V potential (*DC*, vs. GND) as the reference input potential (*V_in_*). This potential was provided using the reference potential generator, a resistance (R=1.000 Ω), and a capacitor (10.0 µF, 100.0 V). These elements were paralleled with the helminth eggs as by-pass capacitance during electrical connections through the two-microelectrode system and the coaxial wires. This system led to accurately estimate the output potential at a fixed time interval between 0.0-1.0 min, according to the following Equation (Eq. 3, SI) ^7^:

$V_{\mathrm{out}}= -\frac{1}{Ri.Cf}\int_{0.00}^{60.00} V_{\mathrm{in}}\mathrm{dt}$Eq. (3)

, where *C_f_* is the equivalent capacitance of the capacitor and the helminth egg. This result is shown in detail in the main manuscript.

**Part 3: *Resistance***

**Resistivity measurements at constant DC potential/current mode for resistivity measurement.** To estimate the resistivity of each helminth egg, a two-microelectrode system was adopted. About this system, two microelectrodes (working and counter) were implanted into the helminth egg tissue with inter-microelectrode distance 0.0124 ± 0.0008 mm (See Part: Surface area- helminth eggs inter-microelectrode distance) and the surface area of each tested helminth egg (See Part: Surface area- Average surface area of the helminth egg) for applying a constant DC potential as large as +100.0 mV (DC, vs. total applied potential) through a potentioastat. Then, the resistance was measured using an external “*Wheatstone-Bridge*” circuit ^8^ using a linear (multi-turn) potentiometer. The current was also measured by using the AVO (multi-) meter. To set the microelectrode system at the Giga ohm sealed conditions, the position of the electrode system was changed along with resistance measurement at the same time. It should be noted that, due to the high mechanical stability of the surface layer of each helminth egg, a reproducible microelectrode implanting process was achieved using the stepper motor modules.

**Part 4: *Surface area***

**The geometric surface area of the microelectrode system**

Due to the smooth surface of the adopted microelectrode system, the geometric surface area of the microelectrode system was measured. For this purpose, the width of the microelectrode was measured using a digital micrometer (Precision: ±0.005 mm). Then, the height of the microelectrode, contacted with the helminth egg was directly measured, based on the number of steps of the stepper motor as well as the degree of the linear movement of another micrometer as the mechanical interface (Figure S2). Finally, the geometric surface area of the cone (i.e., +----+ cylinder) was estimated according to the A=A_Cone_ +A_Cylinder_= (1+5^1/2^) Πr^2^ + 6Πr^2^ formula ^9^. As, the height of the microelectrode was limited to the Giga ohm sealed condition of the helminth egg, consequently, the surface area of the microelectrode was estimated to be 0.00026 (± 0.00001, n= 5) cm^2^.

**Helminth eggs inter-microelectrode distance.** Owing to the high roughness of the external surface of each helminth egg, it was necessary to estimate the distance between the inter-microelectrode. So, in order to achieve this goal, the FE-SEM (Field Emission Scanning Electron Microscope) image of the Fasciola hepatica egg, which has been shown in Figure S2a, and the inter-microelectrode distance was estimated based on the HR-AFM (High Resolution-Atomic Force Microscope) imaging (Figure S2b). Based on the voltage profile imaging (Figure S2c), the average inter-electrode distance was estimated to be 0.0124 ± 0.0008 mm (During 5 sequential estimations). However, it should be noted that this value was partially the same when randomly analyzed different types of helminth eggs. As clearly shown (Figure S2c), at a fixed inter-microelectrode distance, partially, the same path length was detected during the image processing (pixel counting based on the red, green, and blue (*RGB*) components) ^10^ about all types of the tested helminth eggs. This result revealed that, in spite of the high active surface area (high roughness) of the helminth eggs, but the same porosity was measured for the tested helminth eggs.


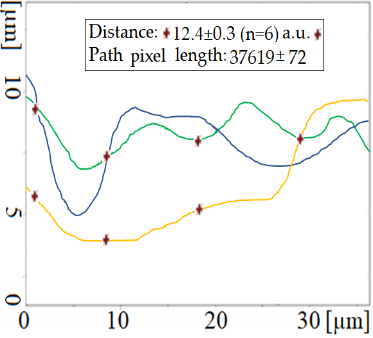

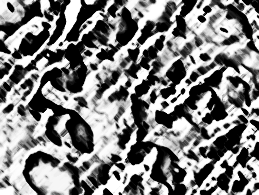

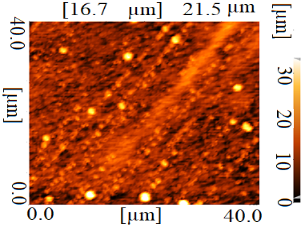


a

b

c

**100 nm**

**Figure S2.** | a) FE-SEM, b) HR-AFM, and c) voltage profile image of Fasciola hepatica egg during estimation of the inter-microelectrode distance. **Condition:** The analysis was achieved on the AC mode on the alumina support.

**The average surface area of the helminth egg**

The average surface area of the helminth egg was also evaluated via estimation of the summation of the multiple longitude voltage profiles on the height profile at whole parts of the HR-AFM image (Figure S3a). For this purpose, the correlation between the voltage profiles and the surface area was evaluated during the calibrations with the Au nanoparticles as reference nanostructures. The high-resolution transmission electron microscopic image has been shown in Figure S3a, synthesized during the reduction of the HAuCl_4_ using ascorbic acid as both reducing and capping agents according to a reported procedure ^11^ with an average diameter of 25 nm.

The average surface area (93.0 ± 0.8 m^2^ g^−1^, n=3) was also estimated by the BET isotherm (Figure S3b). In addition, the horizontal/vertical of the voltage profile parameters of the HR-AFM images about different kinds of helminth eggs and their ratios (based on parts per million, ppm) have been shown in Figure S4. As exhibited, the same ratios were detected during analyses of different types of helminth eggs according to the HR-AFM imaging. At this condition, partially the same values (0.0047 ± 0.0012 cm^2^, n=3) were estimated for each tested egg at a fixed length (0.0124 ± 0.0016 mm, n=3). The reliability of this result was evaluated via estimation of the Au nanoparticles with a relative error percentage of maximum ± 5.0 % (n= 3). The same as the results of the inter-electrode distance, a similar conclusion was again concluded based on the ratio of each horizontal and vertical voltage profile parameters of the HR-AFM images.


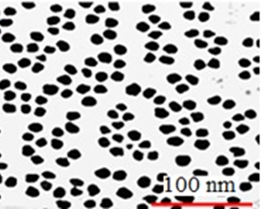


a

**Figure S3.** | a) HR-TEM image and b) BET isotherm of Au nanoparticles used for correlation between the summation voltage profile parameters and surface area at a fixed geometric distance (12.4 µm).

**Extended Data:**

**Figure S4. |** The results of the voltage profile of helminth eggs. **Condition:** Each reported data is the average of three replicate data at different situated of the HR-AFM images under similar conditions.

**References:**

1. Molnar, P.; Hickman, J. J., *Patch-clamp methods and protocols*. Springer Science & Business Media: 2007; Vol. 403.

2. Walz, W.; Boulton, A. A.; Baker, G. B., *Patch-clamp analysis: advanced techniques*. Springer Science & Business Media: 2002.

3. Malboubi, M.; Gu, Y.; Jiang, K., Study of the tip surface morphology of glass micropipettes and its effects on giga-seal formation. In *Electronic Engineering and Computing Technology*, Springer: 2010; pp 609-619.

4. Platt, C., *Encyclopedia of Electronic Components Volume 1: Resistors, Capacitors, Inductors, Switches, Encoders, Relays, Transistors*. " O'Reilly Media, Inc.": 2012; Vol. 1.

5. Halliday, D.; Resnick, R.; Walker, J., Fundamental of Physics 6th Edition. Amerika. John Wiley & Sons, Inc: 2003.

6. Jung, W., *Op Amp applications handbook*. Newnes: 2005.

7. Skoog, D. A.; Holler, F. J.; Crouch, S. R., *Principles of instrumental analysis*. Cengage learning: 2017.

8. Hoffmann, K., *Applying the Wheatstone bridge circuit*. HBM Germany: 1974.

9. Krivoshapko, S. N.; Ivanov, V., *Encyclopedia of analytical surfaces*. Springer: 2015.

10. Lupton, R.; Blanton, M. R.; Fekete, G.; Hogg, D. W.; O’Mullane, W.; Szalay, A.; Wherry, N., Preparing Red‐Green‐Blue Images from CCD Data. *Publications of the Astronomical Society of the Pacific* **2004,** *116* (816), 133.

11. Suchomel, P.; Kvitek, L.; Prucek, R.; Panacek, A.; Halder, A.; Vajda, S.; Zboril, R., Simple size-controlled synthesis of Au nanoparticles and their size-dependent catalytic activity. *Scientific reports* **2018,** *8* (1), 1-11.
